# P-2006. Individuals on Immunosuppressive or Immunomodulatory Therapies Remain at Increased Risk of COVID-19 Hospitalization, Despite Vaccination: Findings from INFORM, a Retrospective Health Database Study in England

**DOI:** 10.1093/ofid/ofae631.2163

**Published:** 2025-01-29

**Authors:** Sabada Dube, Lucy Carty, Carla Talarico, Renata Yokota, Richard McNulty, Michelle Harley, Jurgens Peters, Nahila Justo, Ana Goios, Kathryn Evans, Yi Lu, Sylvia Taylor, Jennifer Quint, Rachael A Evans

**Affiliations:** Medical Evidence, Vaccines and Immune Therapies Unit, AstraZeneca, Cambridge, England, United Kingdom; AstraZeneca, Cambridge, England, United Kingdom; Vaccines and Immune Therapies, BioPharmaceuticals Medical, AstraZeneca, Gaithersburg, MD, USA, Gaithersburg, Maryland; P95, Leuven, Belgium, Dilbeek, Luxembourg, Belgium; Medical Affairs, Vaccines and Immune Therapies Unit, AstraZeneca, Cambridge, UK, Cambridge, England, United Kingdom; AstraZeneca, Cambridge, England, United Kingdom; AstraZeneca, Cambridge, England, United Kingdom; Real-World Evidence, Data Analytics, Evidera, Stockholm, Sweden and Department of Neurobiology, Care Science and Society, Karolinska Institute, Stockholm, Sweden, Stockholm, Sodermanlands Lan, Sweden; P95 Epidemiology & Pharmacovigilance, Leuven, Brabant Wallon, Belgium; Real-World Evidence, Data Analytics, Evidera, Waltham, MA, USA, Waltham, Massachusetts; Real-World Evidence, Data Analytics, Evidera, London, UK, London, England, United Kingdom; Medical Evidence, Vaccines and Immune Therapies Unit, AstraZeneca, Cambridge, UK, Cambridge, England, United Kingdom; National Heart and Lung Institute, Imperial College London, London, UK, Cambridge, England, United Kingdom; University of Leicester, Leicester, England, United Kingdom

## Abstract

**Background:**

Use of immunosuppressive or immunomodulatory therapies (IITs) may be a risk factor for severe COVID-19 outcomes. The INFORM (INvestigation oF cOvid-19 Risk among iMmunocompromised populations) study (ISRCTN53375662) uses routinely collected, national primary/secondary care data in England to provide a comprehensive, population-based assessment of COVID-19 impact, risk, and cost among immunocompromised (IC) and other high-risk populations. Here we assess risk of COVID-19 hospitalization in IC individuals receiving IITs during the Omicron period in 2023.

**Figure:**
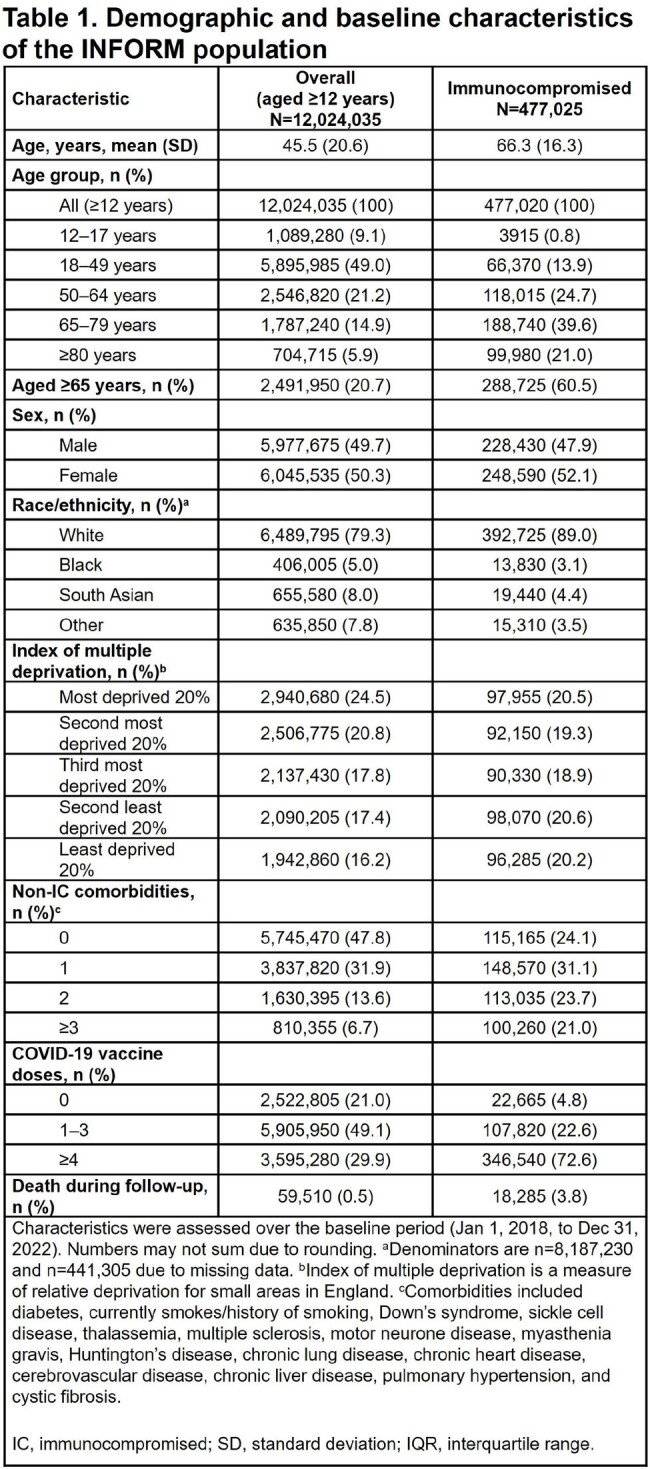

**Methods:**

Retrospective cohort study using pseudonymized electronic health records of a random sample of 25% of the population of England aged ≥ 12 years, registered and active in the NHS from January 1 to June 30, 2023. Crude incidence rates (IRs) per 1000 person-years (PY) and incidence rate ratios adjusted for age, sex, and comorbidities (aIRRs) for COVID-19 hospitalization were estimated for the overall population and those who received ≥ 4 COVID-19 vaccines by January 1, 2023 (fully vaccinated individuals).

**Figure:**
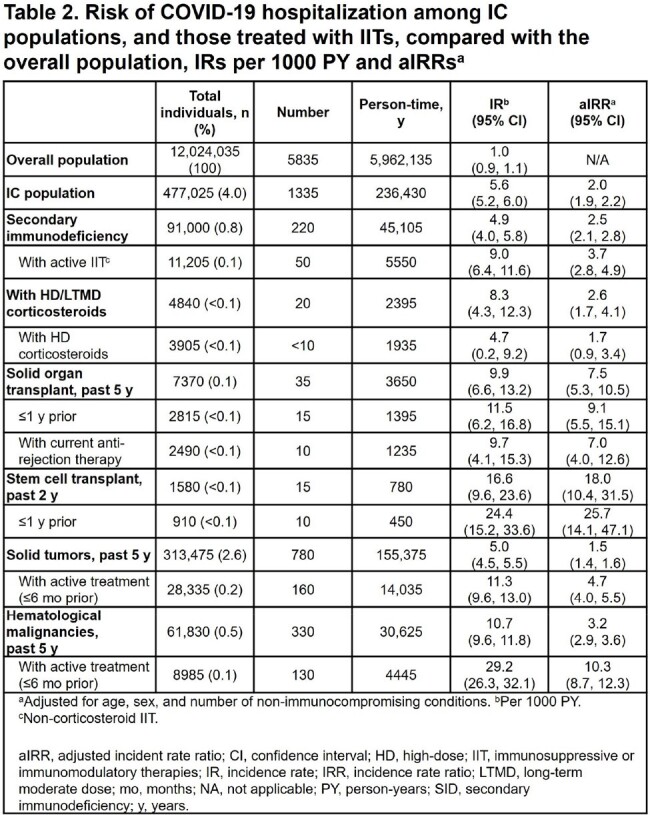

**Results:**

Of the 12,024,035 individuals in this analysis, 477,025 (4.0%) were IC (Table 1). Of the 5835 hospitalizations, 1335/5835 (22.9%) were among IC individuals (Table 2). In fully vaccinated individuals, there were 4135 hospitalizations, with 1010/4135 (24.4%) among IC individuals (Table 3). Recent or active treatment with IITs was associated with higher risk of hospitalization. Among fully vaccinated individuals, secondary immunodeficiency was associated with an aIRR for hospitalization of 2.5 (95% CI: 2.2, 2.9); aIRR for hospitalization for those with secondary immunodeficiency with active IIT was 3.6 (95% CI: 2.6, 4.9). Fully vaccinated individuals with solid tumors and hematological malignancies were associated with aIRRs for hospitalization of 1.4 (95% CI: 1.3, 1.5) and 3.0 (95% CI: 2.6, 3.4), respectively; aIRRs for fully vaccinated individuals with active cancer treatment were 4.0 (95% CI: 3.3, 4.9) and 9.2 (95% CI: 7.4, 11.3), respectively.

**Figure:**
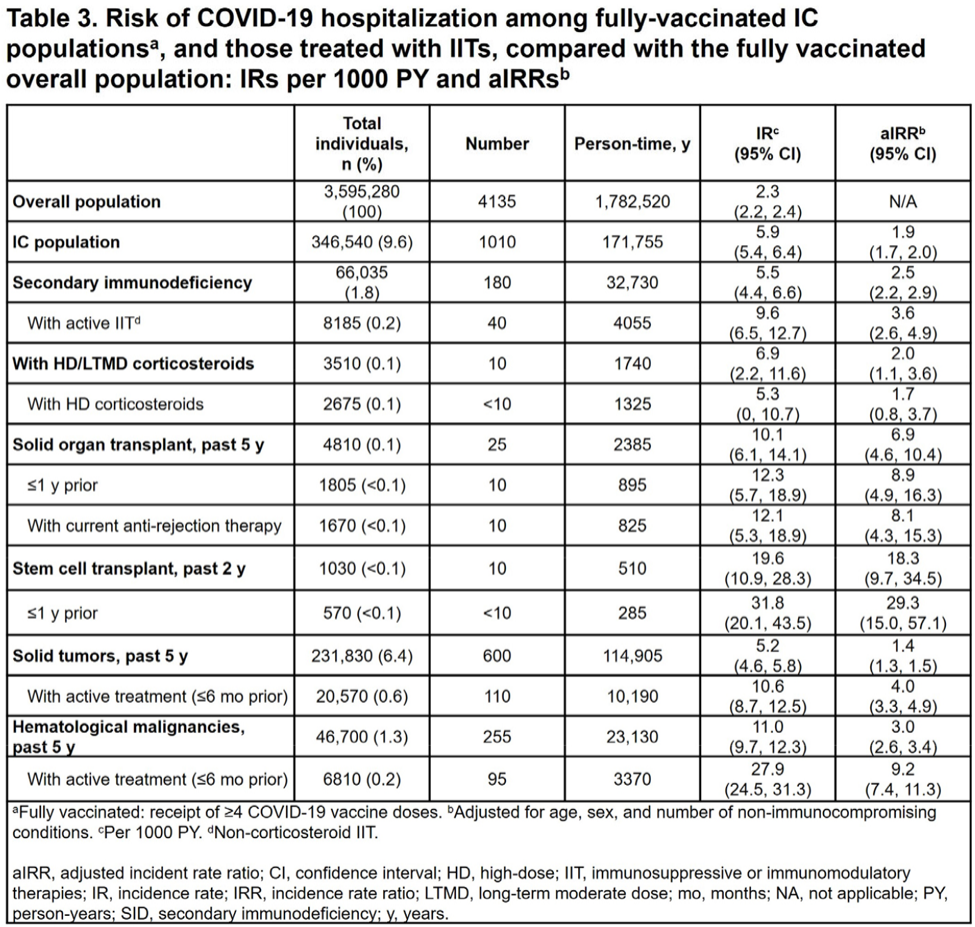

**Conclusion:**

Despite vaccination, IC individuals treated with IIT are at increased risk of COVID-19 hospitalization. Additional COVID-19 preventative measures should be considered for IC individuals, especially for those receiving IIT.

**Disclosures:**

Sabada Dube, PhD, AstraZeneca: Employee of, and may hold stock and/or stock options Lucy Carty, PhD, AstraZeneca: Employee of, and may hold stock and/or stock options Carla Talarico, PhD, MPH, AstraZeneca: Employee of AstraZeneca and may hold stock and/or stock options Renata Yokota, PhD, P95 Epidemiology & Pharmacovigilance/AstraZeneca: Employee of P95 Epidemiology & Pharmacovigilance, which received a consultancy fee for the conduct of the current study from AstraZeneca Richard McNulty, MD, AstraZeneca: Employee of, and may hold stock and/or stock options Michelle Harley, MBBS, MRCP, MRCGP, AstraZeneca: Employee, holds or may hold stock Jurgens Peters, MD, MPH, MSc, MBA, AstraZeneca: Employee, holds or may hold stock Nahila Justo, PhD, MBA, Evidera/AstraZeneca: Employee of Evidera, which received funding for the conduct of the current study from AstraZeneca Ana Goios, PhD, P95 Epidemiology & Pharmacovigilance: Employee of P95 Epidemiology & Pharmacovigilance, which received a consultancy fee for the conduct of the current study from AstraZeneca Kathryn Evans, MPH, Evidera/AstraZeneca: Employee of Evidera, which received funding for the conduct of the current study from AstraZeneca Yi Lu, PhD, Evidera/AstraZeneca: Employee of Evidera, which received funding for the conduct of the current study from AstraZeneca Sylvia Taylor, PhD, MPH, MBA, AstraZeneca: Employee of, and may hold stock and/or stock options in, AstraZeneca Jennifer Quint, PhD, Asthma + Lung UK: Grant/Research Support|AstraZeneca: Advisor/Consultant|AstraZeneca: Grant/Research Support|Boehringer Ingelheim: Grant/Research Support|Evidera: Advisor/Consultant|Evidera: Funding|GSK: Advisor/Consultant|GSK: Grant/Research Support|Health Data Research UK (HDR UK): Grant/Research Support|Insmed: Advisor/Consultant|Medical Research Council (MRC): Grant/Research Support Rachael A. Evans, PhD FRCP, Boehringer Ingelheim: Speaker fees|Chiesi: Support for attending the British Thoracic Society (BTS)|Chiesi: Serving non-paid as the European Respiratory Society (ERS) Group 01.02 Pulmonary Rehabilitation and Chronic Care secretary|Chiesi: Serving non-paid as the American Thoracic Society (ATS) Pulmonary Rehabilitation Assembly chairperson|Evidera: Funding for providing advice throughout the current study|Genetec/Roche: Grant/Research Support|Moderna: Speaker fees|NIHR: Grant/Research Support|UKRI: Grant/Research Support|Wolfson Foundation: Grant/Research Support

